# Outcome of critically ill patients receiving systemic chemotherapy on the intensive care unit

**DOI:** 10.3389/fonc.2024.1508112

**Published:** 2025-01-06

**Authors:** Panagiotis Karagiannis, Felix Klingler, Viktor Arelin, Winfried Alsdorf, Christina König, Kevin Roedl, Walter Fiedler, Katja Weisel, Stefan Kluge, Carsten Bokemeyer, Dominic Wichmann

**Affiliations:** ^1^ Department of Oncology, Hematology and Bone Marrow Transplantation with Section of Pneumology, University Medical Centre Hamburg-Eppendorf, Hamburg, Germany; ^2^ Department of Intensive Care Medicine, University Medical Centre Hamburg-Eppendorf, Hamburg, Germany

**Keywords:** 1 year mortality, survival predictors, chemotherapy, intensive care unit, oncology

## Abstract

**Objective:**

Analyze the outcomes of critically ill patients who developed new-onset organ dysfunction and received systemic chemotherapy during their ICU stay.

**Design:**

Retrospective cohort study.

**Setting:**

A tertiary medical center in Germany with an Intensive Care Medicine department consists of 11 intensive care units comprising 140 beds, serving all subspecialties of adult intensive care medicine.

**Patients:**

167 patients receiving systemic oncological treatment from January 1st, 2015 to December 31st, 2021, with a data cut-off on December 31st, 2022.

**Interventions:**

None.

**Measurements and main results:**

A total of 167 patients were included. The primary reasons for ICU admission were respiratory failure and shock/sepsis, each accounting for 34% of cases, while complications associated with oncological therapy accounted for less than 8%. The median age of hematological patients (n = 129) was 62 years (IQR 50–70), and for solid tumor patients (n = 38), it was 60 years (IQR 52–65). Predominant disease entities included lymphoma (43%) and acute myeloid leukemia (29%) among hematological patients, and lung cancer (47%) and gastrointestinal malignancies (17%) among solid tumor patients. Hematological patients had a significantly higher median Simplified Acute Physiology Score II (47 vs. 39 points; p=0.013), a higher need for invasive mechanical ventilation (59% vs. 50%; p=0.3), renal replacement therapy (54% vs. 24%; p < 0.001), and a higher 1-year mortality rate (64% vs. 53%; p=0.2) compared to solid tumor patients. The hazard ratio for 1 year survival for male sex was 2.34 (1.31–3.49), for mechanical ventilation 2.01 (1.33–3.04), for vasopressor therapy 1.98 (1.27–3.10), and for renal replacement therapy 1.51 (1.03–2.23), respectively.

**Conclusion:**

Administering intravenous chemotherapy in an ICU setting remains challenging, and the experience to establish an indication for systemic chemotherapy is still challenging. However, the study demonstrates that, after careful interdisciplinary decision-making, a substantial number of patients can benefit from it.

## Highlights

- Question: What are the outcomes of critically ill patients who developed new-onset organ dysfunction and received systemic chemotherapy during their ICU stay?
- Findings: Hematologic patients were predominantly male (76%) and had higher SAPS-II scores, requiring more supportive interventions such as invasive ventilation and renal replacement therapy. One-year mortality was 64% for hematologic patients and 53% for solid tumor patients. Lower SAPS-II scores were associated with decreased mortality in solid tumor patients.
- Meaning: Administering intravenous chemotherapy in an ICU setting remains challenging. However, the study demonstrates that, after careful interdisciplinary decision-making, a substantial number of patients can benefit from it.

## Introduction

The outcome of patients with hematological malignancies and with solid tumors has improved continuously over the last decades (1). In addition to the further development of primary therapies, the improved management of complications has also contributed significantly to this success (2, 3). Currently, nearly one in five patients treated in an Intensive Care Unit (ICU) has a cancer diagnosis (4, 5). Numerous studies in recent years have demonstrated that early involvement of intensive care medicine in managing complications is beneficial for patients, particularly in cases of respiratory failure, renal impairment, or chemotherapy-associated complications (6–8).

The integration of intensive care medicine into the treatment regimen for cancer patients has been transformative. Historically, the prognosis for cancer patients requiring ICU admission was poor, largely due to the severity of their underlying disease and the complications arising from both the malignancy and its treatment. However, with advancements in medical technology, critical care practices, and a better understanding of the pathophysiology of cancer and its complications, outcomes have significantly improved. This improvement is evident in the enhanced survival rates and quality of life for these patients.

Recent consensus statements recommend ICU admission for treatment of an acute complication if the life expectancy of the underlying malignancy is more than one year (9). However, there are still insufficient data on whether the primary start of chemotherapy in an intensive care unit for critically ill patients with malignancies also leads to a better outcome (10). A recently published narrative review elaborates on the challenges of systemic chemotherapy in the ICU setting (11). The aim of our study was to analyze the outcome of critically ill patients with a new onset of organ dysfunction and received systemic chemotherapy during this ICU stay.

## Materials and methods

This retrospective analysis was performed at the Department of Intensive Care Medicine at the University Medical Center Hamburg-Eppendorf. During the study period the department consisted of 11 intensive care units comprising of 140 beds serving all subspecialties of adult intensive care medicine. All patients receiving systemic oncological treatment from January 1^st^ 2015 to December 31^th^ 2021 on ICU were included in this study. At the time of data cut-off on December 31^th^ 2022, patient data were censored.

Data was collected through electronical patient data management system (PDMS, Integrated Care Manager^®^ (ICM), Version 9.1 – Draeger Medical, Luebeck, Germany). Data extracted included age, sex, underlying hematologic/oncologic disease, and extracorporeal organ support (mechanical ventilation, renal replacement therapy, vasopressors). Furthermore, laboratory parameters, Simplified Acute Physiology Score (SAPS II) and Therapeutic Intervention Scoring System (TISS) (12–14), and information on ICU/hospital length of stay, 28-day and one-year mortality. Premedication was extracted from the hospital’s electronic prescribing system. Routine laboratory assessment was performed on daily basis according to internal standards. This study was approved by the ethics committee of the chamber of physicians Hamburg Germany (WF-004/21). Informed consent was waived due to the observational character of this study.

### Statistical analysis

Descriptive statistics were used to summarize the data. Continuous variables were described as median (25-75% IQR) and compared between groups using the non-parametric Wilcoxon rank-sum test. Normal distribution was assessed using a D’Agostino’s K-squared test. Categorical variables were described as n (%) and compared between groups using Fisher’s exact test. Survival was calculated by Kaplan-Meier estimation and survival rates were compared using Log rank test. “Diseased status” was used as the primary endpoint of the analysis and survival intervals were calculated from the time of ICU admission to the event, i.e., 28, 60, and 365 days after admission to the ICU; if no event occurs time interval was set to 28, 60 or 365 days, respectively. Patients discharged from the hospital either to a rehabilitation unit outside of the primary hospital or to the outpatient sector were counted as ‘discharged from hospital alive’. Statistical analyses were performed using R (The R Foundation for Statistical Computing, Vienna, Austria) and RStudio (Version 2023.09.0 + 463, RStudio PBC, Boston, USA) with packages ggplot2 (Version 3.4.2), ggpubr (Version 0.6.0), dplyr (Version 1.1.1), readxl (Version 1.4.2; Tidyverse, RStudio PBC, Boston, USA), survival (Version 3.5-5), survminer (Version 0.4.9; Alboukadel Kassambara et al., Open source) officer (Version 0.6.2; David Gohel et al., Open source), gtsummary (Version 1.7.2; Daniel D. Sjoberg, Joseph Larmarange, Michael Curry, Jessica Lavery, Karissa Whiting, Emily C. Zabor), forestmodel (Version 0.6.2; Nick Kennedy) and SPSS Statistics (Version 27, IBM, Armonk, USA). Graphics were then edited and merged in Microsoft Powerpoint (Microsoft, Redmond, USA). A p-value of <0.05 was considered to be statistically significant.

## Results

**Figure 1:**
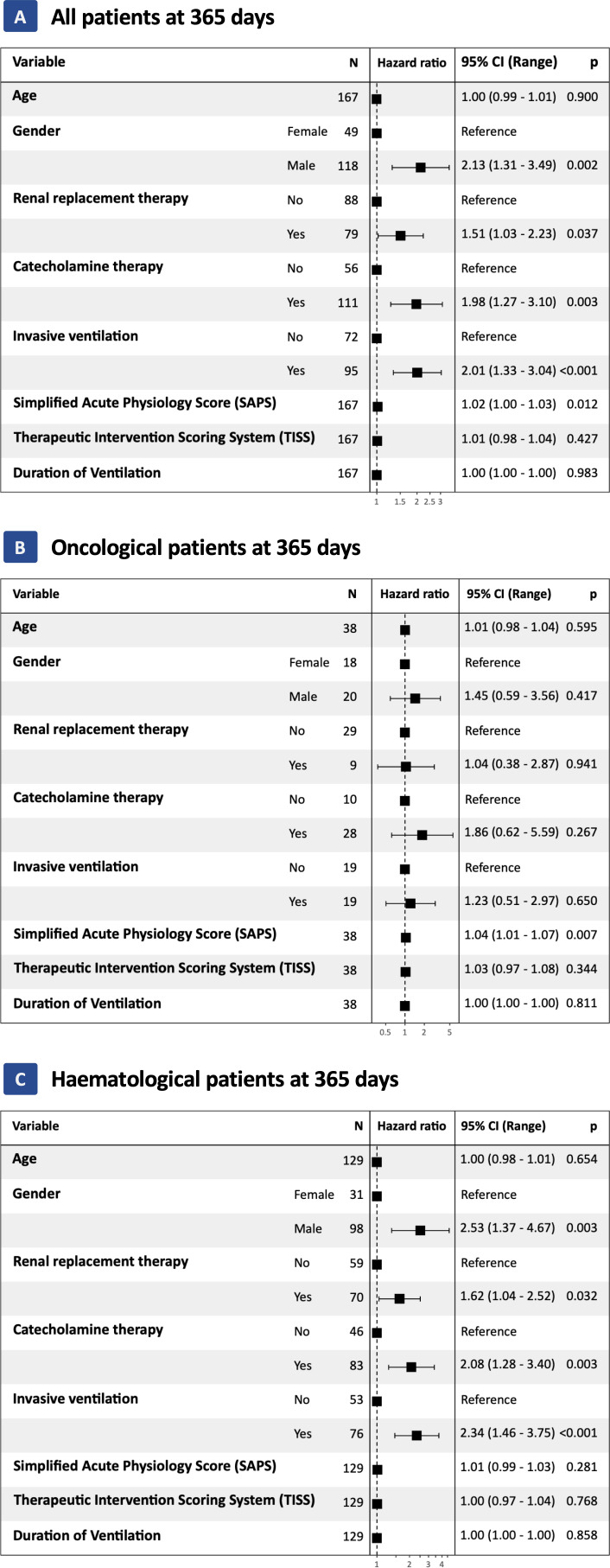
Results from univariate Cox proportional Hazard modeling at 365 days for all patients (Panel **A**), only for Solid tumor patients (Panel **B**) and only for Hematological patients (Panel **C**). Hazard ratios were calculated for the risk of death at 365 days after ICU admission. 95% Confidence interval is shown with lower and upper limits.

**Figure 2:**
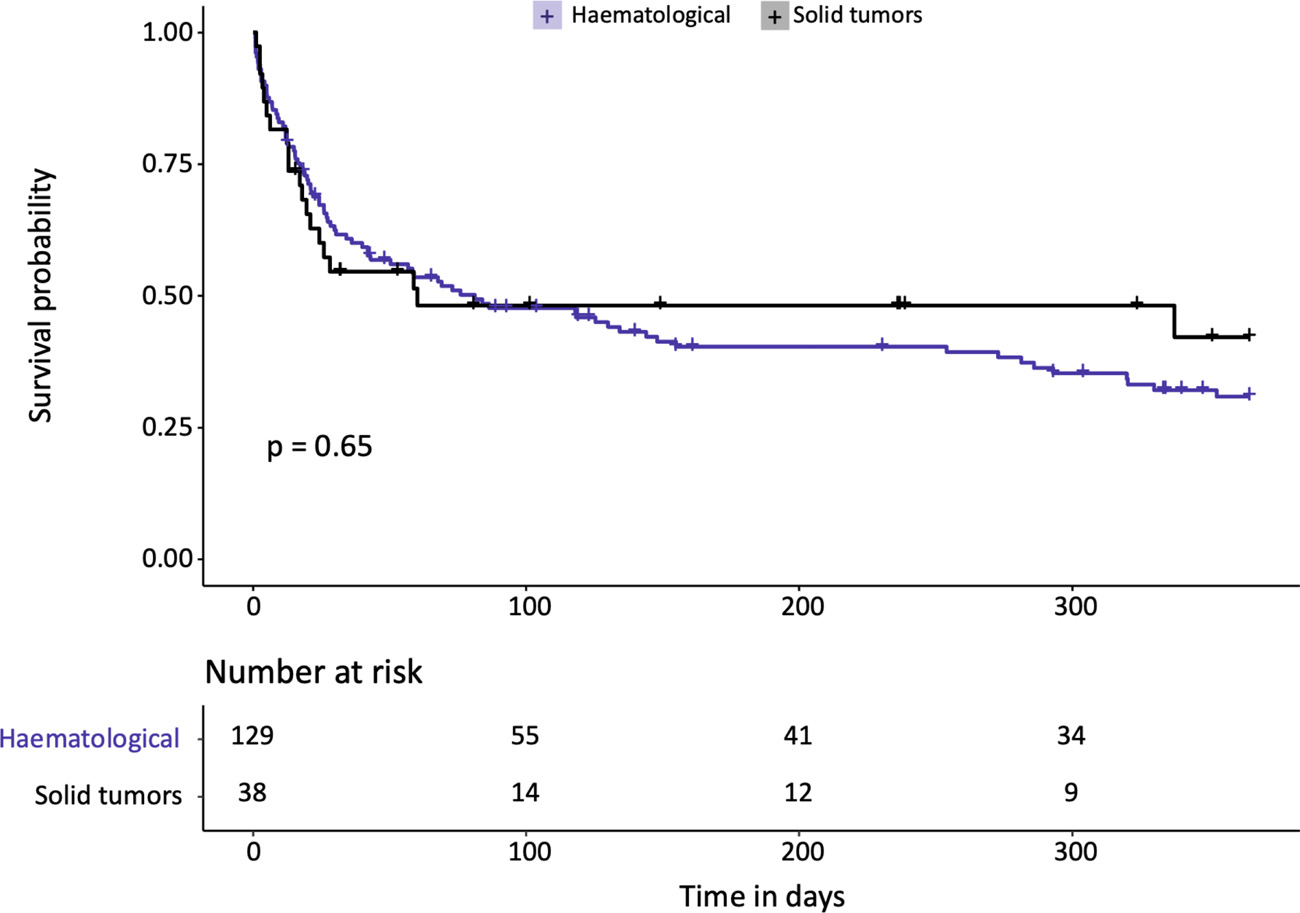
Results of Kaplan-Meier estimates survival analysis by entity group (Hematological, Solid tumors) for an interval of 365 days after ICU admission. Given level of significance is calculated by Log-Rank analysis.

**Figure 3:**
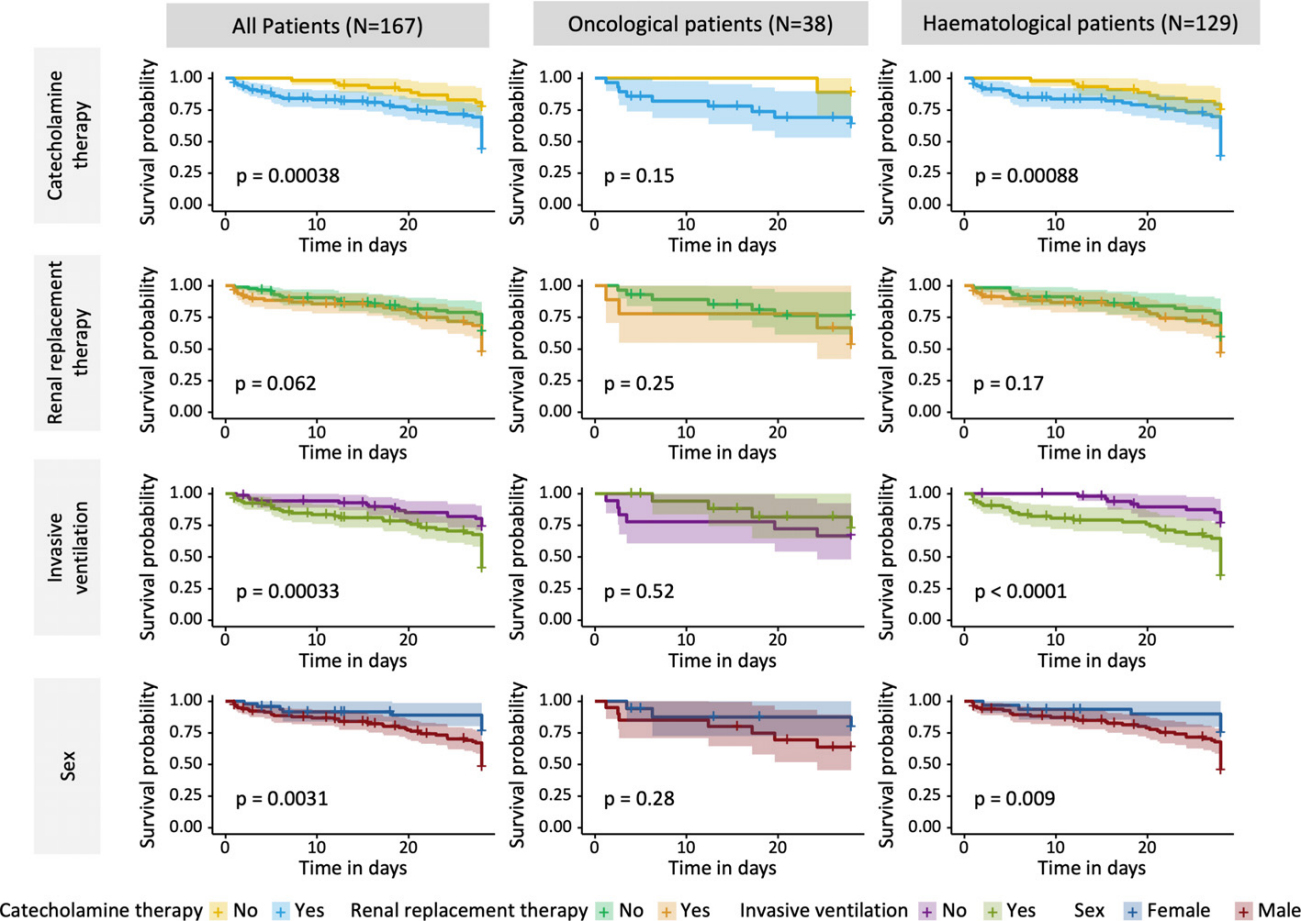
Results of Kaplan-Meier estimates survival analysis by intervention (Catecholamine therapy, Renal replacement therapy, Invasive ventilation) and Sex for an interval of 28 days after ICU admission. Given level of significance is calculated by Log-Rank analysis. 95% Confidence interval is shown by filled light color aside the curves.

One-hundred-sixty-seven patients had malignancies, of which 129 were hematologic and 38 solid tumors. The median age for hematologic and solid tumor patients was 62 years (IQR 50 – 70) and 60 years (52 - 65), respectively. In the hematologic cohort 76% (n=98) were males, whereas sex distribution was more balanced with 53% (n=20) males in the solid tumor cohort. Lymphoma (43%) and acute myeloid leukemia (29%) were the most frequent hematological entities whereas lung cancer (47%) and gastrointestinal malignancies (17%) were the leading solid entities. Respiratory failure, shock/sepsis, altered mental status, renal failure and others including treatment related conditions were the reason for admission to the ICU in 35%, 32%, 12%, 9% and 12% of hematology patients and in 32%, 40%, 13%, 8% and 8% of solid tumor patients. During the treatment course on the ICU, hematologic patients required numerically but not statically significant more often invasive ventilation (59% vs 50%; p=0.3) and renal-replacement therapy (RRT) (54% vs 24%; p<0.001). Hematological patients had a significant higher SAPS-II Score than oncological patients (SAPS-II; median: 47 vs 39 points; p=0.013) and the one-year mortality was 64% (n=83) vs. 53% (n=20), respectively (**Table 1**). However, the overall one-year-survival for all patients was 38%. Risk factors for one-year mortality in hematological patients were invasive mechanical ventilation (HR 2.34; 95%CI 1.46 – 3.75; p<0.001), male sex (HR 2.53; 95%CI 1.37 – 4.67; p=0.003), catecholamine therapy (HR 2.08; 95%CI 1.28 – 3.40; p=0.003) and renal replacement therapy (HR 1.62; 95%CI 1.04 – 2.52; p=0.032) (**Figure 1**). In solid tumor patients, a higher SAPS II was statistically associated with decreased survival (HR 1.04; 95%CI 1.01 – 1.07; p<0.007). The ICU-mortality was 42% in both groups (**Table 1**). One-year mortality in hematological patients was 64% and 53% in solid tumor patients (p = 0.65; **Figure 2**). Individual risk factors were calculated and are illustrated as Kaplan-Maier graphs for an interval of 365 days after ICU admission in **Supplementary Figure 1**, for an interval of 28 days after ICU admission in **Figure 3** and for an interval of 60 days after ICU admission in **Supplementary Figure 4**. In this context, risk factors are similar for all intervals. The decision on chemotherapy was made by the interdisciplinary team consisting of ICU physicians and cancer specialists. The aim was to use chemotherapy as part of the multimodal approach to stabilize the underlying organ dysfunction related (in part) to the underlying malignancy. Palliative therapy that did not serve this purpose was paused until the patient had stabilized. Overall, about half of the patients started chemotherapy after ICU admission (54% and 63% in heamtological and Oncological patients, respectively). Due to the heterogeneity of the underlying disease entities, a large number of substances and combinations were used (**Supplementary Table 1**). The proportion of patients treated with either monotherapy or combination therapy did not differ when stratified by low (<2 organs) or high degree (≥ 2 organs) of organ dysfunction (**Supplementary Figure 2**). However, survival rates stratified 365 days after ICU admission was statistically significant better in patients only needed none or one organ system support (HR 2.36; 95%CI 1.48 - 3.75; p < 0.001) which is shown in **Supplementary Figure S3**. The rate of patients discharged from the hospital alive was 42% in the hematological and 45% in the oncological patients cohort, respectively.

**Table 1:** Demographic data and cancer diagnosis of patients receiving systemic oncological therapy on the intensive care unit (ICU).

Variable	Hematological Patients N = 129		Oncological Patients N = 38	
**Demographic data**	Median (IQR) or N [%]		Median (IQR) or N [%]	
Age	62 (50 – 70)		60 (52 – 65)	
Male Sex	98 [76 %]		20 [53 %]	
TISS	10 (5 – 15)		10 (5 – 14)	
SAPS	47 (37 – 55)		39 (28 – 50)	
**Entities** (N)				
	Lymphoma	56	Lung Cancer	18
	AML	38	GI Cancer	8
	Multiple Myeloma	15	Gynecological Cancer	3
	Hemophagocytosis	10	Prostate/Urothelial/Germ cell	4
	ALL	6	Sarcoma	2
	CML	2	CUP	2
			Glioblastoma	1
	Missing	2	Missing	0
**Supportive therapy**	N [%]		N [%]	
Vasopressor therapy	83 [64 %]		28 [74 %]	
Invasive ventilation	76 [59 %]		19 [50 %]	
Renal replacement therapy	70 [54 %]		9 [24 %]	
**Follow up parameters**	N [%]		N [%]	
Length of ICU stay Median (IQR)	10 (5 – 22)		6 (4 – 20)	
Hospital discharge alive N [%]	54 [42 %]		17 [45 %]	
ICU discharge alive N [%]	75 [58 %]		22 [58 %]	
Follow up time Mean (95 % CI)	260 (175 - 345)		171 (86 - 256)	
One year mortality	64 %		53 %	
Started Chemotherapy on ICU	70 [54 %]		24 [63 %]	
Missing data N	49		10	

## Discussion

In our cohort of 167 ICU patients with hematological and solid cancers, systemic chemotherapy in addition to treatment of acute organ dysfunctions resulted in a one-year overall survival of 38%. More and more evidence has emerged that early ICU treatment of therapy-related complications is beneficial for patients with malignancies (2, 6, 15). In contrast to critical illness of non-cancer related disease the outcome of patients with malignancy is not alone depending on the antecedent condition but also on the type and stage of cancer. Therefore, it is necessary to treat not only the antecedent critical condition but also the underlying tumor. Hence, it is not surprising that in our study we could demonstrate that patients admitted to the ICU because of acute organ dysfunction also benefit from the initiation of systemic tumor therapy. This held true not only for hematological patients with tumor-related organ dysfunction where systemic chemotherapy is a therapeutic option under certain conditions (11, 16, 17), but also for oncological patients where higher grade organ dysfunctions is usually considered a contraindication for such therapies (18). We demonstrated in 167 patients with acute organ dysfunction a one-year mortality of 62%. This is similar to the range reported in other studies in patients with sepsis or septic shock without a cancer diagnosis (19–21). For non-cancer patients with acute respiratory failure the one-year-mortality rate has been reported to be 31 – 48% (22, 23) and for patients with acute kidney failure 32 – 62% (24, 25). Respiratory failure is also the risk factor associated with the highest mortality in non-cancer patients. Nevertheless, there are substantial differentials with regard to the underlying cause as Secreto and colleagues (26) showed recently in a *post-hoc* analysis of the EFRAIM study. Moreover, we were able to show that 43% (n=71) of the patients treated, could be discharged from hospital alive, indicating that treatment even in patients with higher grade organ dysfunction can lead to acceptable quality of life within the expected range for these disease entities (27). Risk factors (respiratory failure requiring mechanical ventilation, male sex) were comparable hazards to those in patients without active cancer. Daily bedside interdisciplinary meetings between intensive care physicians and oncologists allowed adaptation of the cancer therapy to the patient`s current health condition and thus management of side effects. In addition to this the ICU staff was trained to provide both, application of chemotherapy and critical care. The high degree of organ dysfunction in our cohort made it impossible to detect chemotherapy-associated adverse events. Due to the retrospective character of this study a clinical bias for patient selection and therapy limitation cannot be excluded. Moreover, the rather low number of patients and monocentric recruitment limits the generalizability of the study results. Further, this study primarily focuses on combination chemotherapy regimens and does therefore not address single-agent targeted therapies, which are generally associated with a more favourable toxicity profile. On the other hand, a prospective study investigating the withholding of potentially life-saving chemotherapy would be difficult for obvious ethical reasons. As previously reported by Darmon et al. (28) survival for these patients has improved over the past decade and outdated evidence indicating a treatment limitation needs to be addressed to update the clinician’s’ perspective on critically ill cancer patient. From this point, our studies demonstrated that both withholding specific therapy and limiting intensive care measures do not seem appropriate in this collective. Moreover, the addition chemotherapy *may extend survival, providing patients with valuable time to engage with their family members*. Based on our retrospective data, we have developed a decision-making algorithm (**Supplementary Figure S5**) for interdisciplinary ICU teams.

## Conclusion

The initiation of systemic chemotherapy for patients with acute organ dysfunctions who are being treated in an ICU and have newly diagnosed oncological or hematological cancers appears to be both beneficial and feasible. To optimize outcomes for individual patients, a high level of interdisciplinary collaboration is essential.

## Data Availability

The raw data supporting the conclusions of this article will be made available by the authors, without undue reservation.
