# Supplementary material for: Outcome of critically ill patients receiving systemic chemotherapy on the intensive care unit
Source: Front Oncol. 2025 Jan 6;14:1508112. doi: 10.3389/fonc.2024.1508112 (PMC11743169; doi:10.3389/fonc.2024.1508112)
Supplement: Supplementary file 1 [file DataSheet1.pdf]

Supplementary Material

Figure Titles

Supplement Figure 1 – Results of Kaplan-Meier estimates survival analysis by intervention (Catecholamine therapy, Renal replacement therapy, Invasive ventilation) and Sex for an interval of 365 days after ICU admission. Given level of significance is calculated by Log-Rank analysis. 95% Confidence interval is shown by filled light color aside the curves.

Supplement Figure 2 – Frequency plot depicting absolute count for Number of invasive supported organ systems, Complexity of tumor therapy and status at one year after ICU admission. Colored connection lines between groups show multiple characteristics for patients in the respective group of Status at 1 year.

Supplement Figure S3 – Results of Kaplan-Meier estimates survival analysis by number of supported Organ systems for an interval of 365 days after ICU admission. Given level of significance is calculated by Log-Rank analysis.

Supplement Figure S4 – Results of Kaplan-Meier estimates survival analysis by intervention (Catecholamine therapy, Renal replacement therapy, Invasive ventilation) and Sex for an interval of 60 days after ICU admission. Given level of significance is calculated by Log-Rank analysis. 95% Confidence interval is shown by filled light color aside the curves.

Supplement Figure S5 – Suggested flow chart of clinical decision making for patients with malignant diseases.

Table Titles

Supplement Table S1 – Tumor therapy regimens. Abbreviations: ALL: Acute lymphoblastic leukemia; ATRA: all-trans retinoic acid; BEACOPP: Cyclophosphamide + Doxorubicine + Etoposide + Procarbazine + Prednisolone + Vincristine + Bleomycine; CHO(E)P: Cyclophosphamide + Doxorubicine + Vincristine + Prednisolone (+ Etoposide); DA(C): Daunorubicine + Cytarabine (+ Cladribine); DHAC: Cytarabine + Carboplatin + Dexamethasone; HiPEC: Hyperthermic Intraperitoneal Chemotherapy; MEC: Mitoxantrone + Etoposide + Cytarabine; ICE: Ifosphamide + Etoposide + Carboplatin.

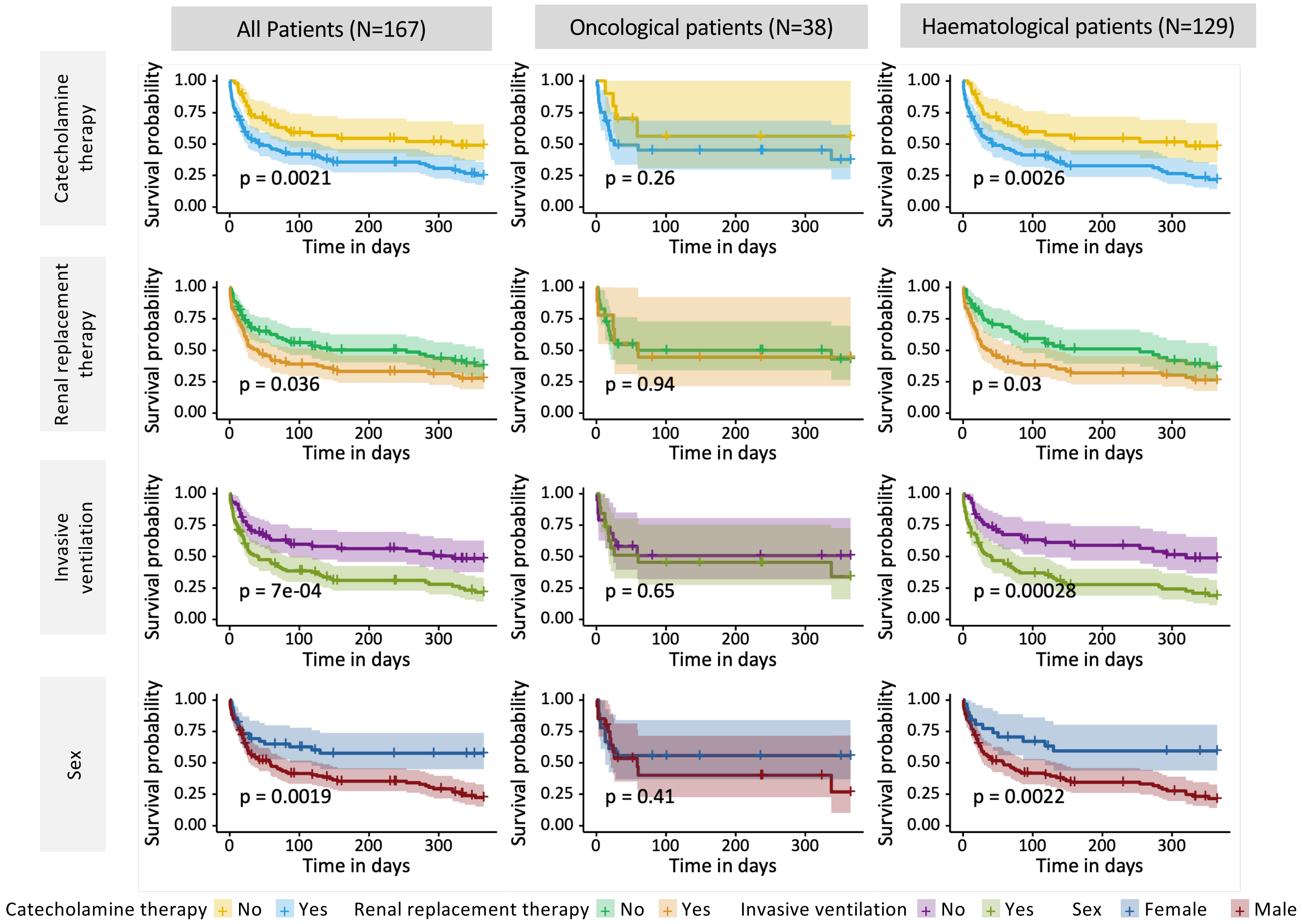

Supplement Figure S1 – Results of Kaplan-Meier estimates survival analysis by intervention (Catecholamine therapy, Renal replacement therapy, Invasive ventilation) and Sex for an interval of 365 days after ICU admission. Given level of significance is calculated by Log-Rank analysis. 95% Confidence interval is shown by filled light color aside the curves.

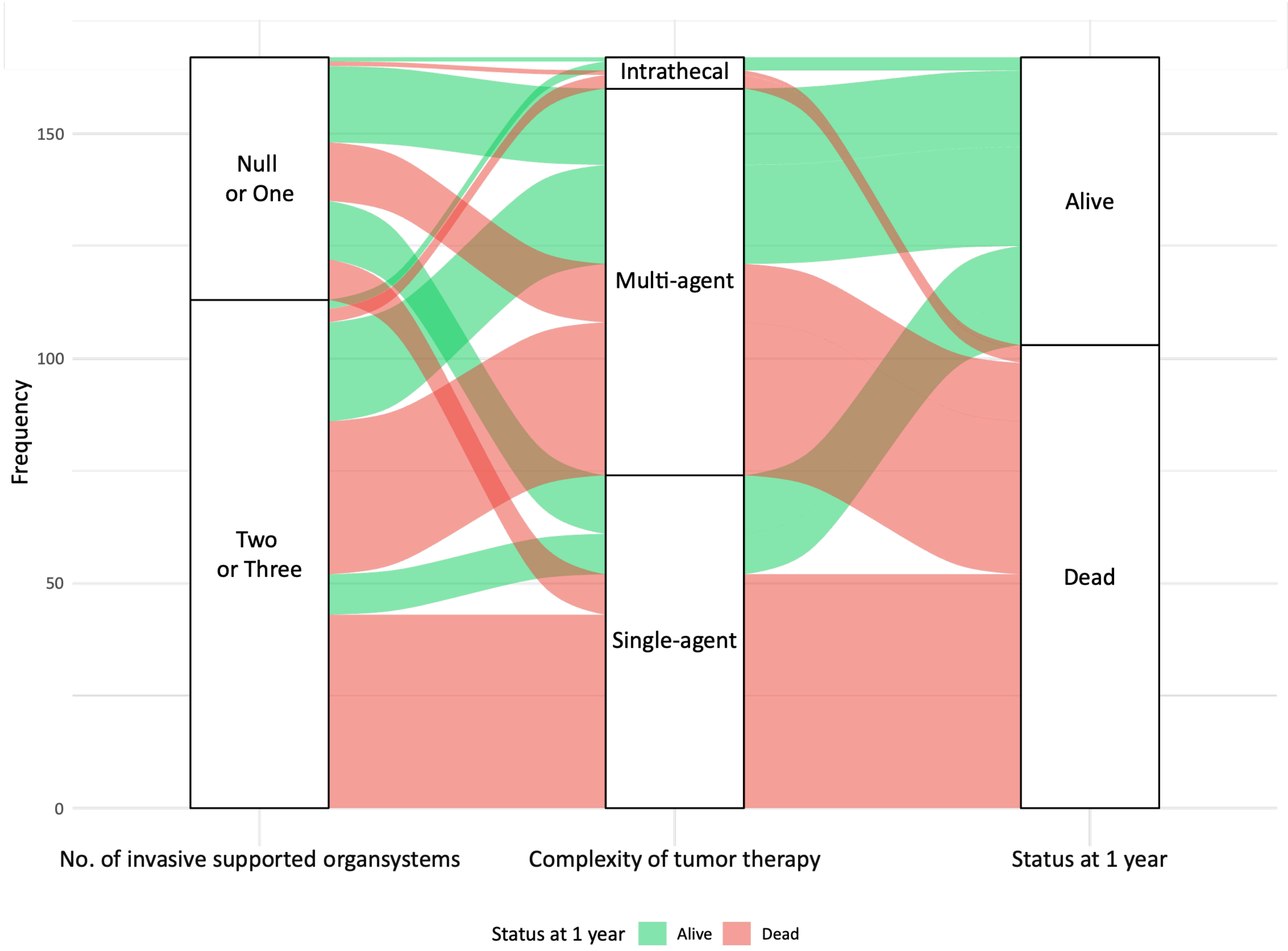

Supplement Figure S2 – Frequency plot depicting absolute count for Number of invasive supported organsystems, Complexity of tumor therapy and Status at 1 year after ICU admission. Colored connection lines between groups show multiple characteristics for patients in the respective group of Status at 1 year.

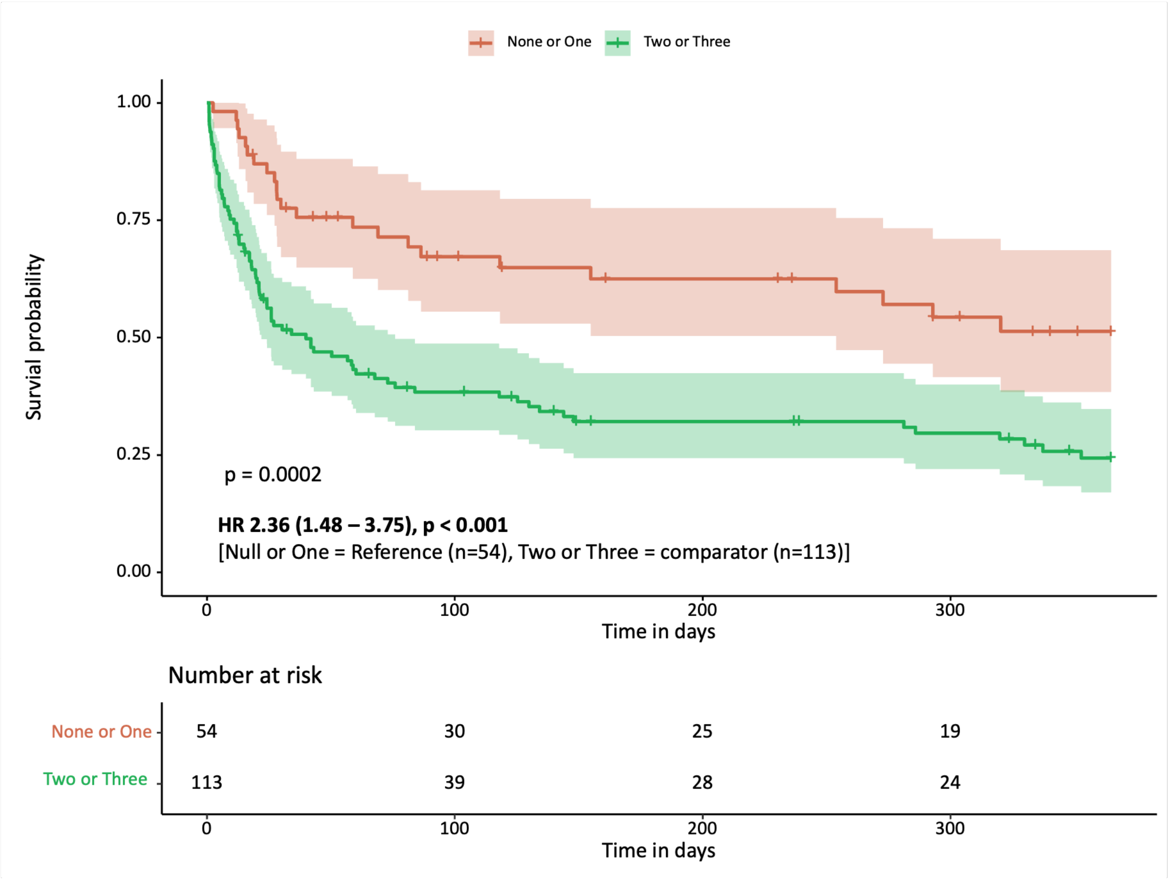

Supplement Figure S3 – Results of Kaplan-Meier estimates survival analysis by number of supported Organ systems for an interval of 365 days after ICU admission. Given level of significance is calculated by Log-Rank analysis.

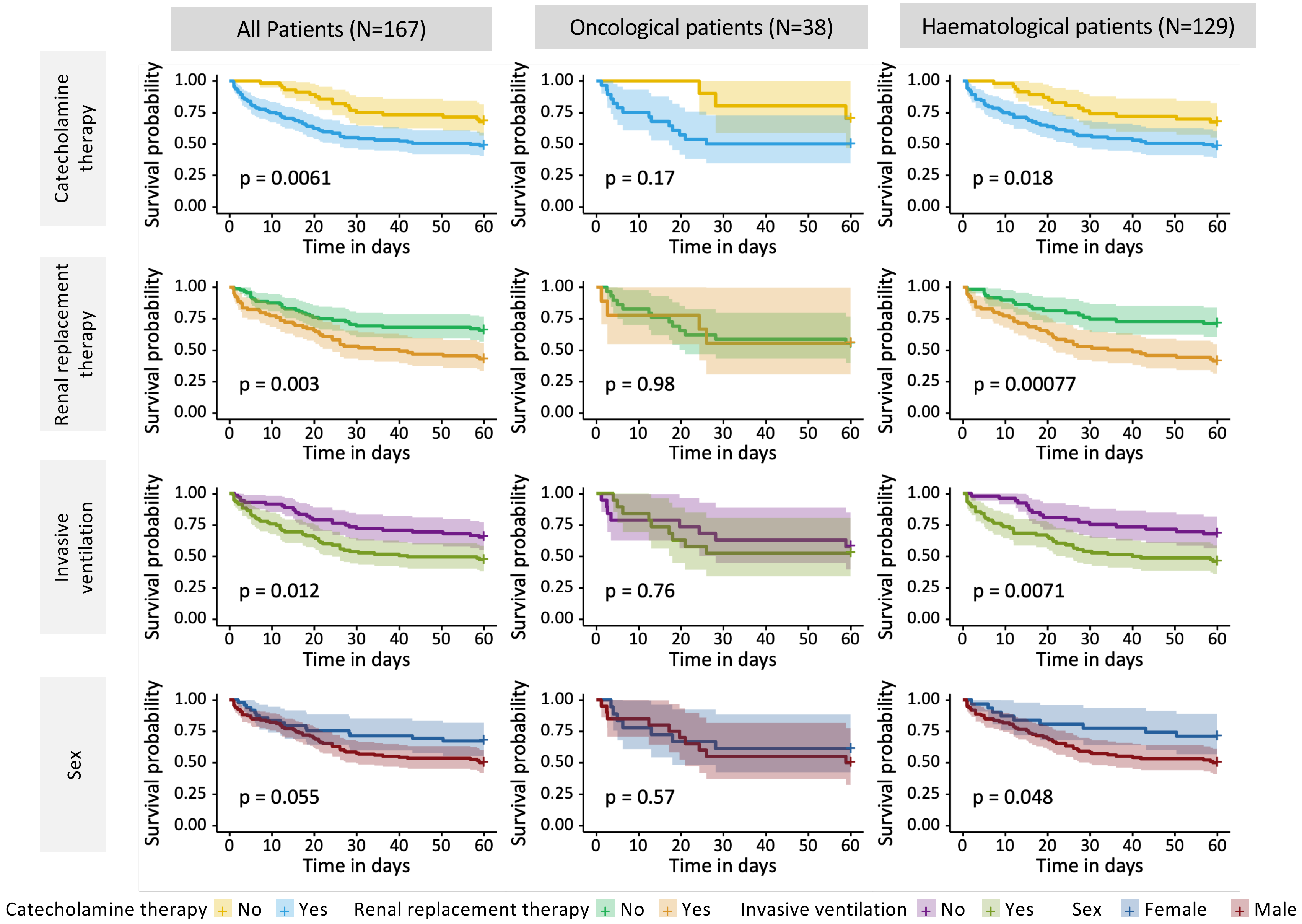

Supplement Figure S4 – Results of Kaplan-Meier estimates survival analysis by intervention (Catecholamine therapy, Renal replacement therapy, Invasive ventilation) and Sex for an interval of 60 days after ICU admission. Given level of significance is calculated by Log-Rank analysis. 95% Confidence interval is shown by filled light color aside the curves.

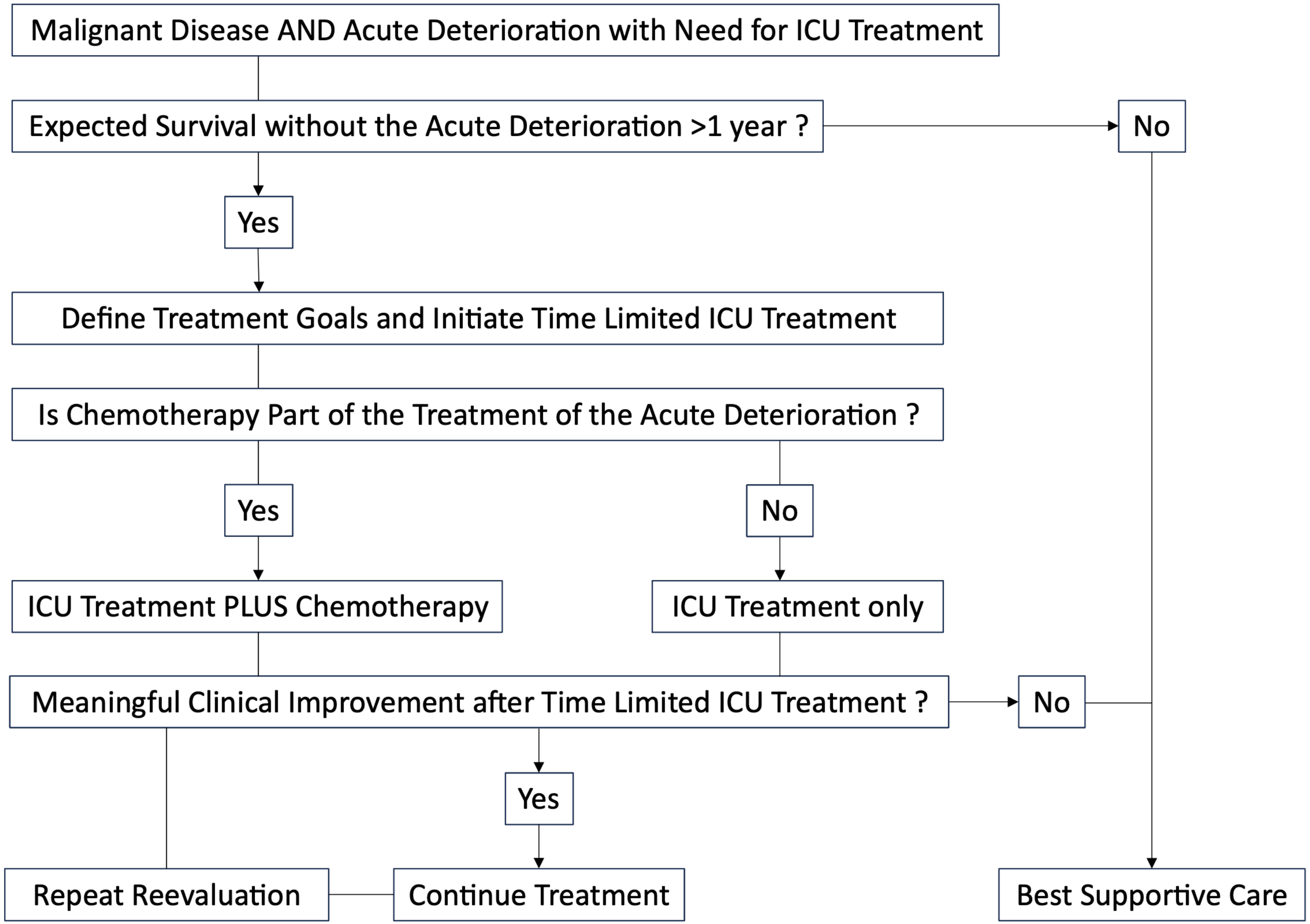

Supplement Figure S5 – Suggested flow chart of clinical decision making for patients with malignant diseases.

**Tumor therapy regimen**	**No. of Patients treated**, N [%]
Patients treated with immunotherapy	34 (20%)
All Regimens	
Alemtuzumab-mono	4 (2.4%)
ALL-therapy	1 (0.6%)
ATRA + Idarubicin	2 (1.2%)
ATRA-mono	1 (0.6%)
Azacitidine + Venetoclax	7 (4.2%)
Azacitidine-mono	1 (0.6%)
BEACOPP	1 (0.6%)
Blinatumomab	1 (0.6%)
Bortezomib + Dexamethasone	19 (11%)
Brentuximab-vedotine	2 (1.2%)
CHOEP	2 (1.2%)
CHOP	4 (2.4%)
Cyclophosphamide + Cytarabine	1 (0.6%)
Cyclophosphamide + Prednisolone	9 (5.4%)
Cyclophosphamide + Vincristine	1 (0.6%)
Cytarabine + Ifosphamide	1 (0.6%)
Cytarabine-mono	7 (4.2%)
DA	10 (6.0%)
DAC	3 (1.8%)
Decitabine	2 (1.2%)
DHAC	1 (0.6%)
Doxorubicine + Ifosphamide	1 (0.6%)
Doxorubicine-mono	3 (1.8%)
Eribulin-mono	1 (0.6%)
Etoposide + Vincristine	1 (0.6%)
Etoposide-mono	10 (6.0%)
Fludarabine-mono	2 (1.2%)
Fluorouracil	1 (0.6%)
Fluorouracil + Oxaliplatin	1 (0.6%)
High-dose Methotrexate	2 (1.2%)
HiPEC	3 (1.8%)
Irinotecan-mono	1 (0.6%)
MEC	2 (1.2%)
Nivolumab	1 (0.6%)
Obinutuzumab-Ibrutinib	1 (0.6%)
Only intrathecal therapy	7 (4.2%)
Platin-based therapy	21 (13%)
Polatuzumab-vedotine	1 (0.6%)
Rituximab + CHOP	3 (1.8%)
Rituximab + DHAC	2 (1.2%)

Rituximab + Etoposide	1 (0.6%)
Rituximab + ICE	1 (0.6%)
Rituximab + Thiothepa	2 (1.2%)
Rituximab-mono	14 (8.4%)
Taxol-based therapy	3 (1.8%)
Temozolomide	1 (0.6%)
None	1 (0.6%)

Supplement Table S1 – Tumor therapy regimens. *Abbreviations: ALL: Acute lymphoblastic leukemia; ATRA: all-trans retinoic acid; BEACOPP: Cyclophosphamide + Doxorubicine + Etoposide + Procarbazine + Prednisolone + Vincristine + Bleomycine; CHO(E)P: Cyclophosphamide + Doxorubicine + Vincristine + Prednisolone (+ Etoposide); DA(C): Daunorubicine + Cytarabine (+ Cladribine); DHAC: Cytarabine + Carboplatin + Dexamethasone; HiPEC: Hyperthermic Intraperitoneal Chemotherapy; MEC: Mitoxantrone + Etoposide + Cytarabine; ICE: Ifosphamide + Etoposide + Carboplatin.*
